# Effects of UV-B radiation on epiphytic bacterial communities on male and female *Sargassum thunbergii*

**DOI:** 10.1038/s41598-022-26494-3

**Published:** 2023-03-09

**Authors:** Jing Wang, Zhibo Yang, Peiyao Lu, Yan Sun, Song Xue, Xuexi Tang, Hui Xiao

**Affiliations:** 1 College of Marine Life Sciences, Ocean University of China, Qingdao, 266003 China; 2 Laboratory for Marine Ecology and Environmental Science, Qingdao National Laboratory for Marine Science and Technology, Qingdao, 266000 China

**Keywords:** Biodiversity, Microbial biooceanography, Microbial ecology, Microbial ecology, Environmental impact, Marine biology

## Abstract

The effects of increased UV-B radiation on macroalgae have been widely studied, but knowledge concerning the response of communities of algal epiphytic bacteria to increased UV-B radiation and differences between male and female algae is still lacking. Via 16S rDNA high-throughput sequencing technology, changes in the epiphytic bacterial communities on male and female *S. thunbergii* under increased UV-B radiation were studied in the lab. Under different UV-B radiation intensities, although the α diversity and community composition of epiphytic bacteria changed little, the β diversity indicated that the community structure of bacteria on *S. thunbergii* was obviously clustered, and the relative abundance of dominant bacteria and indicator species changed considerably. There were unique bacteria in each experimental group, and the bacteria whose abundance obviously changed were members of groups related to environmental resistance or adaptability. The variation in the abundance of epiphytic bacteria was different in male and female *S. thunbergii*, and the bacteria whose abundance greatly changed were mainly related to algal growth and metabolism. The abundance of genes with predicted functions related to metabolism, genetic information processing, environmental adaptation and infectious diseases changed with increased UV-B radiation, and those variations differed between epiphytic bacteria on male and female *S. thunbergii*. This study found that the algal epiphytic bacteria were influenced by the increase in UV-B radiation and underwent certain adaptations through adjustments to community structure and function, and this response was also affected by the sex of the macroalgae. These results are expected to serve as experimental basis and provide reference for further understanding of the response of algae epiphytic bacteria to enhanced UV-B radiation caused by the thinning of the ozone layer and the resulting changes in the relationship between algae and bacteria, which may change the community of the marine ecosystem and affect important marine ecological process.

## Introduction

The increase in UV-B (280–315 nm) radiation caused by the continuous depletion of the ozone layer is considered a serious environmental issue. As important primary producers in marine ecosystems and as the organisms that respond mostly directly to increased UV-B radiation, intertidal macroalgae are periodically exposed to air with tidal changes and are highly susceptible to increased UV-B radiation^1^. Studies on macroalgae have shown that increased UV-B radiation can affect macroalgae both macroscopically (age structure, population size and population growth dynamics, etc.)^2,3^ and microscopically (photosynthetic pigment destruction, decrease in photosynthetic rate and reduced glutathione, etc.)^3,4^. In fact, within an appropriate range, there are some positive effects of UV- B enhancement on plant growth and development, such as the regulation of plant morphological development^5^, as well as improving quality by altering the accumulation of sugars and secondary metabolites. It can also induce plants to resist the stress of pathogenic organisms. These effects also happen on macroalgae. UV-B has been found to promote the growth of algae and improve antioxidant defenses, etc.^6,7^. However, the high UV-B radiation presents mainly harmful effects on macroalgae. Studies on *Ulva pertusa*^8^, *Sargassum horner*^9^, and *S. thunbergii*^10–13^ have obtained similar results, including damage to growth and development, decrease in biomass productivity, decline in oxidation and metabolism levels, inhibition of photosynthesis^6,13–16^, etc.

However, most of the relevant research has focused on the effects of increased UV-B radiation on algae itself, there are few studies on epiphytic bacteria of algae. The epiphytic bacteria, which adhere to the surface of marine macroalga, have multifaceted and complicated interactions with their host macroalgae^17^. Algae and bacteria are the major biological factors driving carbon fixation and storage in the ocean. When seaweeds are stressed by enhanced UV-B radiation, the algal epiphytic bacteria are the most direct responders^18^. UV-B radiation enhancement could change the composition and activity of the bacterial community then affect the growth and development of the host algae, resulting in the changes of interaction between bacteria and algae, which will finally influence the marine biogeochemical cycle (such as carbon cycle). The effects of UV-B radiation on algal epiphytic bacteria mostly concerns two scopes. Firstly, UV-B radiation has a direct impact on plant epiphytic bacteria. Studies on higher plants show that different epiphytic bacterial groups have different sensitivities to UV-B radiation, that the growth of bacteria with low resistance is inhibited or that they die, and that the abundance of bacteria with strong resistance increases. For example, the community composition of epiphytic bacteria of the plant *Erigeron breviscapus* and 13 other plant species on the Qinghai-Tibetan Plateau was found to change under UV-B radiation^19,20^. The only study on algal epiphytic bacteria showed that UV-B radiation can affect the community diversity and evenness and the relative abundance of epiphytic bacteria on the red seaweed *Gelidium lingulatum*^21^. Secondly, by affecting algae, UV-B radiation can indirectly affect epiphytic bacteria. Algae and bacteria in the ocean are inseparable and interact intimately^22^. The community structure and composition of algal epiphytic bacteria, the element of which are significantly affected by environmental factors, are to the host state more than those of planktonic bacteria are^22,23^. The exposure to UV-B radiation affected the host algae and then the epiphytic bacteria change accordingly. For example, changes in soluble sugars, free amino acids and soluble proteins in *E. breviscapus* were shown to directly lead to a decrease in the number of epiphytic bacteria and altered bacterial dominance^19^. on this phenomenon in algae. How these indirect effects from the abovementioned changes in *S. thunbergii* and the direct effects of UV-B radiation on epiphytic bacteria alter the bacterial community is investigated in this study.

Recent studies have shown that changes in plants under UV-B radiation differ between sexes. Xu et al.^24^ pointed out that the basal diameter and leaf nitrogen content of male *Populus cathayana* were significantly higher than those of female *P. cathayana*, indicating greater resistance to UV-B radiation in the former. Similarly, when UV-B radiation was increased, the chlorophyll content and osmotic regulatory ability of male mulberry seedlings were higher than those of female mulberry seedlings^25^. However, female *Populus tremula* (L.) seedlings maintained a higher concentration of low-molecular-weight phenolics throughout the experimental period under increased UV-B radiation^26^. There are also sex-related differences in the response of *S. thunbergii* to increased UV-B radiation. Lu et al.^12^ found that with an increase in UV-B radiation, the chloroplast lamellar structure of female *S. thunbergii* was damaged, and the damage was greater than that to male plants. Sun et al. found that the changes in the physiological responses and metabonomics^10^, as well as the carbon cycle^11^ of male and female *S. thunbergii* under UV-B radiation were related to sex. Whether different sexes of algae show different responses to UV-B radiation leads to differences in the response of epiphytic bacterial communities between male and female *S. thunbergii* is also investigated in this study.

*S. thunbergii* is a representative species of dioecious macroalgae, which has gradually become valuable, for it can be used as bait for sea cucumber and abalone^27^. *S. thunbergii*, a very important genus to maintain the stability of the structure and function in coastal ecosystem^28^, plays an important role in nutrient regulation and habitat restoration^29^. Dioecious macroalgae not only are important components of coastal ecosystems but also are important dominant species in many algal ecosystems. In recent years, the application of high-throughput sequencing, which allows for high resolution typing and study of bacteria at the strain level^30^, has gained increased attention for its use in studying the influence of environmental factors on the microbiota^31^. In this study, 16S rDNA high-throughput sequencing technology was used to analyse changes in the epiphytic bacterial communities of *S. thunbergii* under increased UV-B radiation and differences between male and female *S. thunbergii.* This research provides new ideas for the application of high-throughput sequencing technology in the response of epiphytic bacteria on macroalgae to global climate change and has value in theory and in practice for explaining the changes and the host–bacterium relationship of epiphytic bacteria in marine ecosystems, especially macroalgae, in response to increased UV-B radiation.

## Results

3263 different Amplicon sequence variant (ASV)s clustered together (male, 2830 ASVs; female, 2808 ASVs). Good's coverage of all samples was higher than 0.99, and the rarefaction curves of all the samples tended to saturate with increased sequence amount (Supplementary Fig. S1), indicating that the sequencing depth could cover most species of the samples and could be used for further data analysis.

### Effects of increased UV-B radiation on the α diversity and β diversity of epiphytic bacteria on *S. thunbergii*

After the algae were exposed to increased UV-B radiation, there was no statistically significant difference in the α diversity of the epiphytic bacterial community on *S. thunbergii* (*P* > 0.05) (Supplementary Fig. S2), which was slightly lower in the UV-B radiation treatment group than in the control group. There was no statistically significant difference in α diversity between the males and females (*P* > 0.05) (Supplementary Table S1), but there was a significant difference in epiphytic bacteria on female *S. thunbergii* in response to the high and low UV-B radiation intensity treatments (*P* < 0.05) (Supplementary Table S1).

**Figure 1:**
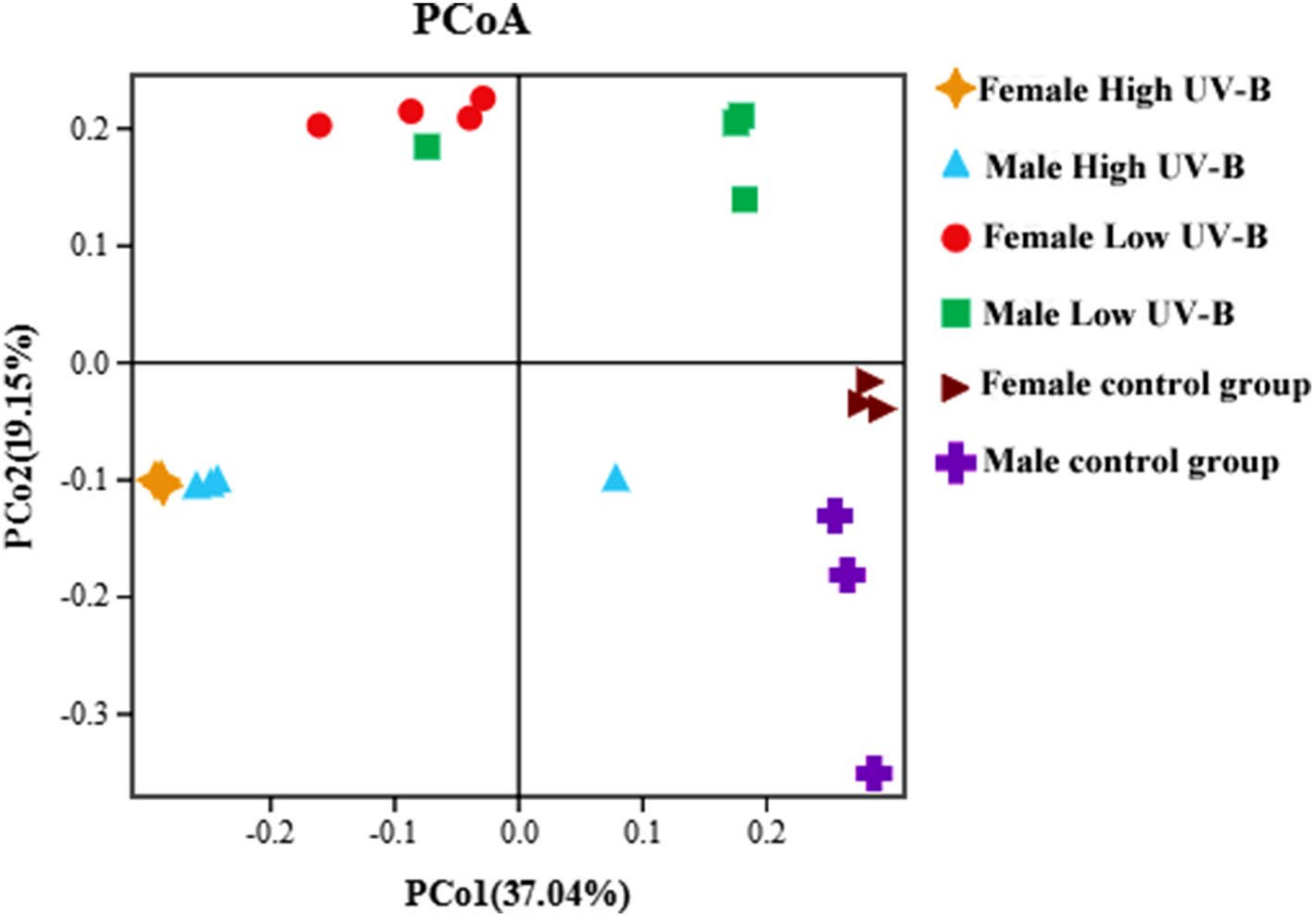
Principal coordinate analysis (PCoA) results of the epiphytic bacterial community on *S. thunbergii* under increased UV-B radiation.

With an increase in UV-B radiation, the epiphytic bacteria of *S. thunbergii* clustered differently (Fig. 1), and there were significant differences in β diversity among the experimental groups (*P* < 0.01) (Supplementary Table S2). Under the same UV-B radiation level, the bacterial communities on the males and females clustered on the basis of the sex of *S. thunbergii*, and there was a significant difference among the groups (*P* < 0.01). Interestingly, the β diversity of the epiphytic bacterial community significantly differed on female *S. thunbergii* (*P* < 0.01) but not on male *S. thunbergii* (*P* > 0.05).

### Effects of increased UV-B radiation on the epiphytic bacteria on *S. thunbergii*

#### Effects of increased UV-B radiation on the community composition of epiphytic bacteria on *S. thunbergii*

**Figure 2:**
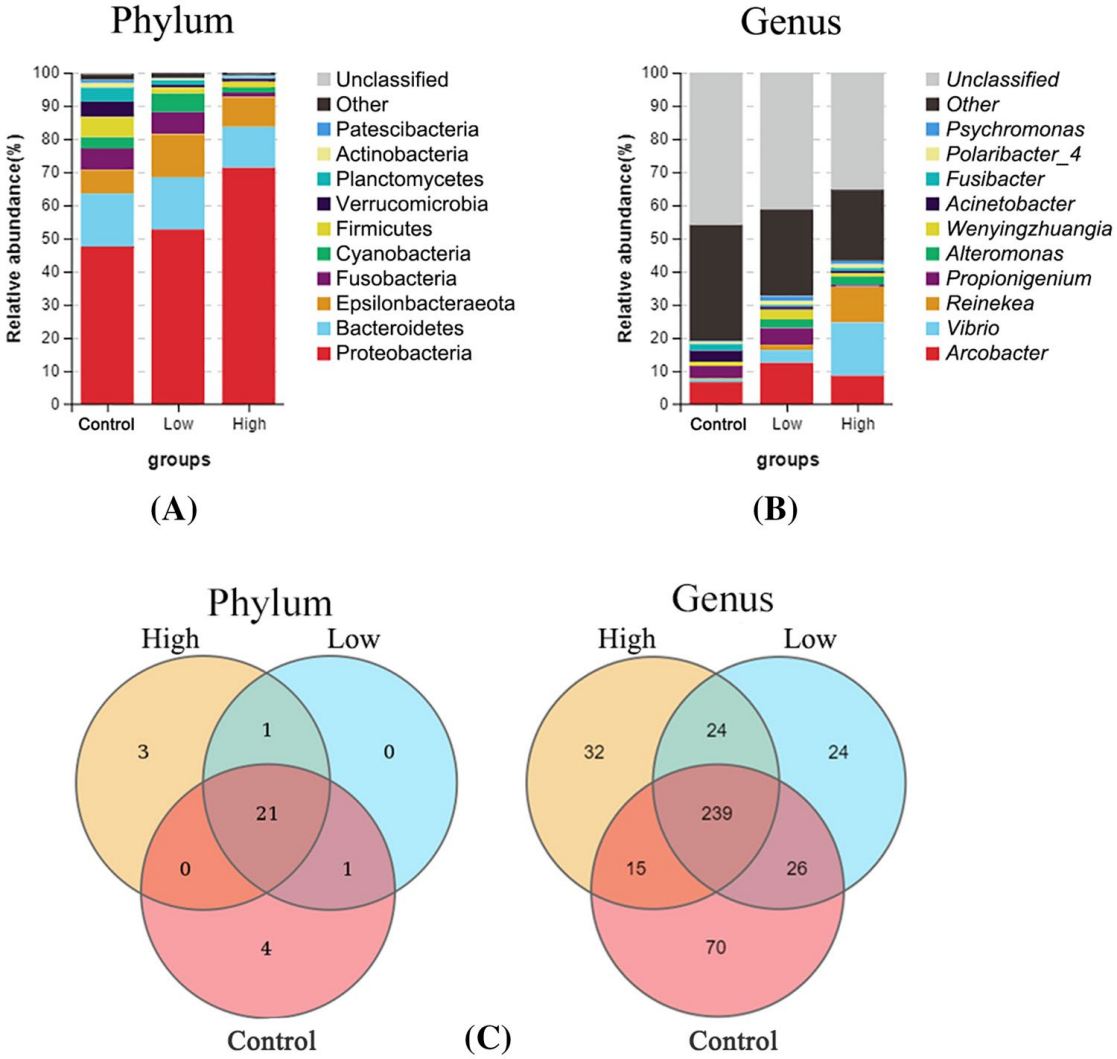
Effects of increased UV-B radiation on the top 10 phyla (**A**) and top 10 genera (**B**) of the epiphytic bacteria of *S. thunbergii* and the top 10 phyla; (**C**) Indicator species (*P* < 0.05) at the phylum level under increased UV-B radiation.

With increased UV-B radiation, the community composition changed little at the phylum level and at the genus level (*P* < 0.05). However, UV-B radiation had a significant effect on the relative abundance of dominant bacteria in the community of epiphytic bacteria on *S. thunbergii* (Fig. 2) (*P* < 0.05).

In total, 30 phyla, 60 classes, and 430 genera of bacteria were detected. Figure 2A showed the dominant phyla of bacteria on *S. thunbergii*, accounting for 98.77–99.70% of all ASVs (Supplementary Table S3), and the remaining phyla of bacteria were classified as “others” or remained unclassified. Proteobacteria was the most dominant bacteria on *S. thunbergii*, followed by Bacteroidetes and Epsilonbacteraeota. Compared with that in the control group, the abundance of some bacterial phyla in the treatment groups changed significantly (*P* < 0.05). The relative abundance of Proteobacteria increased significantly after UV-B radiation treatment (*P* < 0.01)—from 47.60% in the control group to 52.71% under low UV-B radiation and then to 71.31% under UV-B high radiation. Conversely, the relative abundance of Bacteroidetes decreased significantly (*P* < 0.05)—from 15.94% in the control group to 15.73% under low radiation and 12.35% under high radiation. The abundance of Planctomycetes decreases significantly at low radiation (*P* < 0.05) and also at high radiation (*P* < 0.05), While the abundance of Epsilonbacteraeota has no significant change under low and high radiation. The kind of dominant bacteria phyla did not change before and after UV-B radiation treatment, and among the epiphytic bacteria on *S. thunbergii*, Proteobacteria were always the most abundant.

Figure 2B showed the dominant bacterial genera on *S. thunbergii*, accounting for 13.41–25.40% of all ASVs (Supplementary Table S4), and the remaining genera of bacteria were classified as “others” or remained unclassified. Under increased UV-B radiation, the total proportion of the top 12 dominant genera of the epiphytic bacteria on *S. thunbergii* increased significantly (*P* < 0.05)—from 19.41% in the control group to 35.05% under low UV-B radiation and 44.96% under high UV-B radiation. The relative abundance of several dominant bacteria increased significantly (*P* < 0.01); for example, the relative abundance of *Vibrio* increased from 0.95% in the control group to 3.80% under low UV-B radiation and 16.00% under high UV-B radiation, and that of *Reinekea* increased from 0.31% in the control group to 1.62% under low UV-B radiation and 10.74% under high UV-B radiation. However, the abundance of *Propionigenium* decreases significantly (*P* < 0.05) under high radiation. and the abundance of *Polaribacter_4* has no significant change under low and high radiation. After UV-B radiation treatment, the type of dominant bacteria genera did not change, but the relative abundance changed significantly, and genus with the highest relative abundance of bacteria changed from *Arcobacter* to *Vibrio*.

#### Effects of increased UV-B radiation on unique epiphytic bacteria on *S. thunbergii*

During the increased UV-B radiation treatment, the presence of most of the bacterial species did not change. However, the number of species in the treatment group was lower than that in the control group, and there were unique bacteria present under different UV-B intensities.

During the increase in UV-B radiation, the presence of 21 phyla and 239 genera of bacteria did not change (Fig. 2C). The number of unique bacteria on *S. thunbergii* decreased under the low-radiation-intensity treatment but increased under the high-radiation-intensity treatment. For example, at the phylum level, the number of unique bacteria decreased from 4 in the control group to 0 in the low-dose group and then increased to 3 in the high-dose group. The same was true at the genus level − 70 in the control group to 24 in the low-dose group and then to 32 in the high-dose group. The number of bacteria is changing all the time. The difference between the bacteria under high radiation and the control group is large, and there are fewer bacteria in both groups.

#### Effects of increased UV-B radiation on indicator taxa on *S. thunbergii*

**Figure 3:**
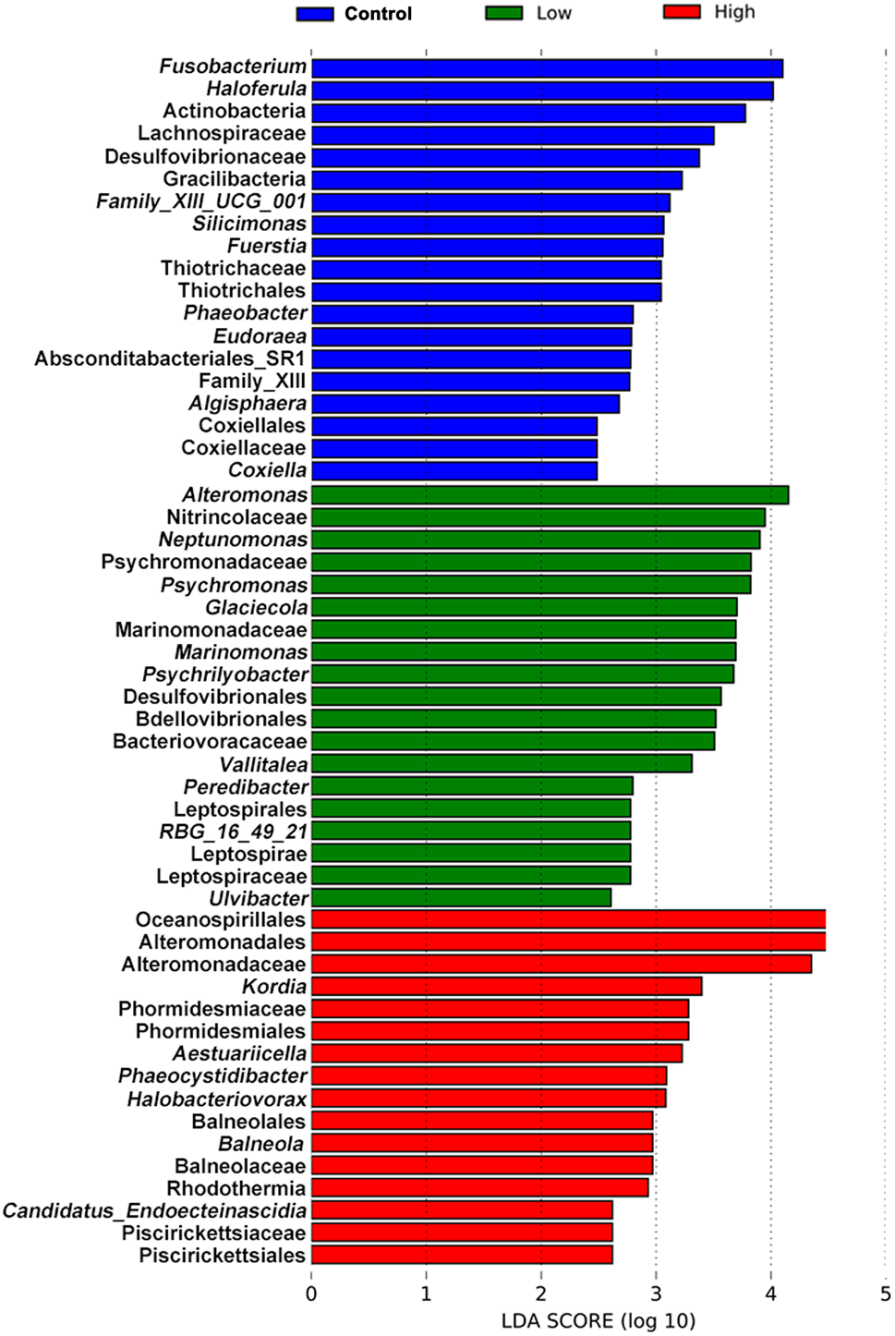
Bacterial species with significant differences in abundance on *S. thunbergii* under increased UV-B radiation (Forest plot showing taxa whose abundance was significantly different between the control group (blue), low-UV-B radiation group (green) and high UV-B-radiation group (red), as determined using the KW test. LDA score (effect size) indicating significant differences in bacterial taxon abundance (*P* value: Wilcoxon rank-sum test, LDA score > 2.0).

**Figure 4:**
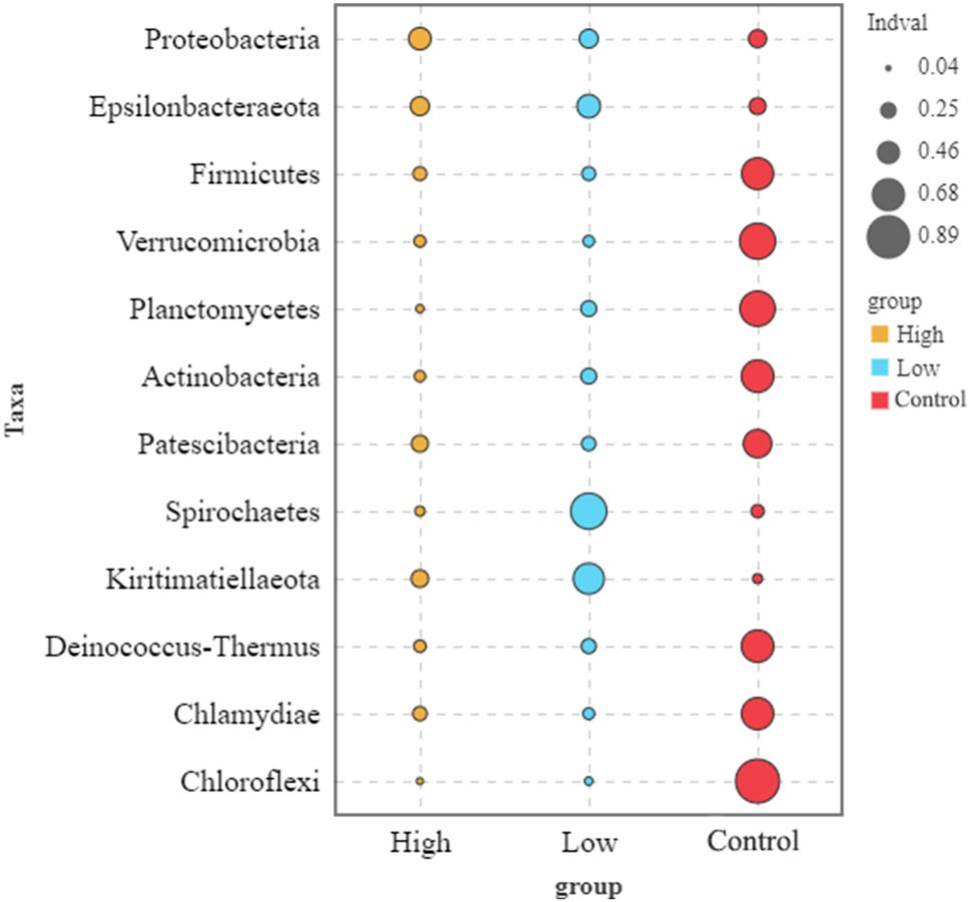
Indicator species (*P* < 0.05) at the phylum level on *S. thunbergii* under increased UV-B radiation. Indicator analysis calculates the indicator value (IndVal) for each species in each group based on the abundance and frequency of occurrence of the species in the sample, with higher values indicating a greater likelihood of the species being an indicator species for that group.

LEfSe analysis and indicator analysis were used to determine the effects of UV-B radiation on the indicator bacteria and indicator values of dominant bacterial phyla (Fig. 3). Indicators with significant differences in abundance were found in all the experimental groups (Fig. 3). With an increase in UV-B radiation, the indicator values of bacteria on *S. thunbergii* tended to be similar, and the differences in the values decreased (Fig. 4).

Each experimental group had its own significantly different indicators. Bacteria of the genera *Fusobacterium* and *Haloferula*, *Bacillus bogoriensis*, Lachnospiraceae, and Desulfovibrionaceae were significantly enriched in the control group; bacteria of the families Nitrincolaceae and Alteromonadales and the genera *Alteromonas* and *Psychrilyobacter* were significantly different under low UV-B radiation (Fig. 3); and bacteria of the orders Oceanospirillales, Alteromonadales and Phormidesmiales and the genus *Kordia* were significantly different under high UV-B radiation.

Under increased UV-B radiation, the indicator types of dominant bacteria at the phylum level gradually decreased. We used indicator species analysis to describe the value of different species for indicating environmental conditions (Fig. 4), and the significantly different bacteria in the high-UV-B radiation treatment group were significantly less than those in the low-UV-B radiation treatment group. In addition, the indicator value of the UV-B treatment group tended to decrease.

### Effects of increased UV-B radiation on the epiphytic bacteria on *S. thunbergii* in different sexes

#### Effects of increased UV-B radiation on the community composition of epiphytic bacteria on *S. thunbergii* in different sexes

**Figure 5:**
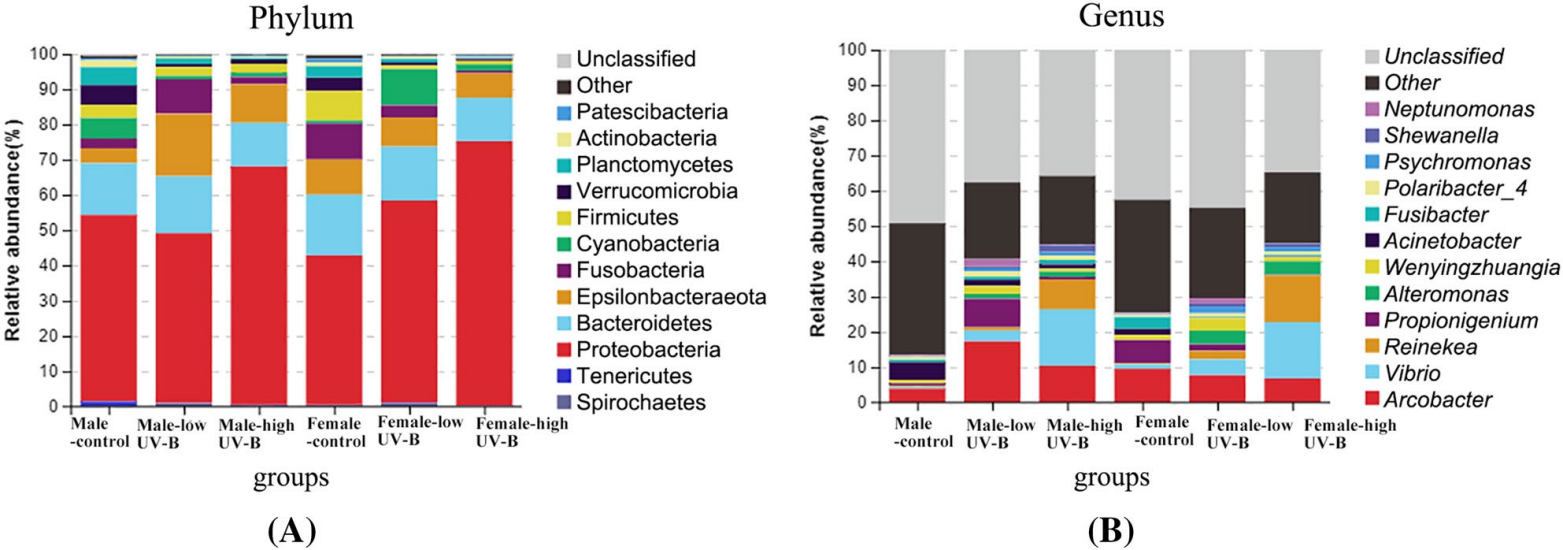
Effects of increased UV-B radiation on the top 12 phyla (**A**) and top 12 genera (**B**) of the epiphytic bacteria on the *S. thunbergii* males and females.

In this study, the change trend of the relative abundance of dominant bacteria between the males and females was not the same (Fig. 5). The total number of phyla, classes, and genera common to both algal sex were 24 phyla, 45 classes and 292 genera (Supplementary Table S5). 28 phyla, 58 classes and 405 genera of epiphytic bacteria were detected on male *S. thunbergii*, while 31 phyla, 58 classes and 389 genera of epiphytic bacteria were detected on female *S. thunbergii*. When UV-B radiation was increased, the abundance of epiphytic bacteria of 7 of the top 12 phyla on males and females exhibited different trends, and the value of the reduction varied (see Table S3 for detailed changes). Interestingly, the five dominant bacterial genera showed different trends on male and female *S. thunbergii*. For instance, from control to low to high UV-B treatments, the abundance of Proteobacteria (52.85%) first decreased (48.12%) and then increased (67.63%) on male *S. thunbergii* but continued to increase (from 42.34% to 57.30% to 74.99%) on female *S. thunbergii*. The abundance of Bacteroides, which had the second largest proportion (14.66%), first increased (16.16%) and then decreased (12.37%) on male *S. thunbergii* but continued to decrease on female *S. thunbergii* (from 17.23% to 15.29% to 12.31%). The trends of the abundance of the other 5 of the top 12 phyla are similar, for example, for Planctomycetes (from 5.13% to 1.59% to 0.45% on males; from 3.27% to 1.06% to 0.32% on females). At the genus level, the change trends of most dominant bacteria were different. The increase or decrease in abundance of *Arcobacter, Propionigenium*, *Wenyingzhuangia, Psychromonas*, *Alteromonas*, *Acinetobacter*, *Fusibacter* and *Polaribacter* was not consistent between male and female *S. thunbergii*, with the exception of the abundance of *Vibrio* (from 0.59% to 3.11% to 16.02% on males; from 1.30% to 4.48% to 15.99% on females) and *Reinekea* (from 0.32% to 0.84% to 8.34% on males; from 0.29% to 2.40% to 13.13% on females) on both male and female *S. thunbergii* (see Table S4 for detailed changes).

#### Effects of increased UV-B radiation on unique epiphytic bacteria on *S. thunbergii* in different sexes

**Figure 6:**
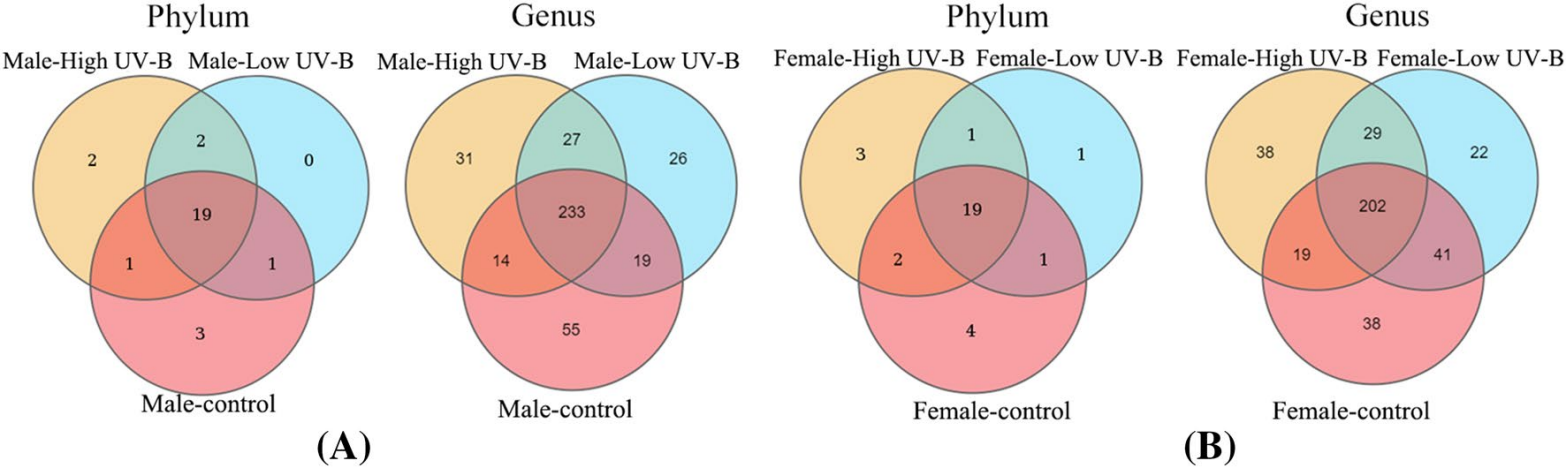
Venn diagram of epiphytic bacteria of *S. thunbergii* under increased UV-B radiation, (**A**) male *S. thunbergii*, and (**B**) female *S. thunbergii* (A mean ASV tag number less than 1 in a group indicates that there were no ASV species in that group).

The changes in unique phyla and genera of bacteria were also different between the different sexes. On the different sexes of *S. thunbergii*, the species of bacteria were not identical (Fig. 6A, B). The 18 of the 19 phyla remained the same on male and female *S. thunbergii*, with the difference being the presence of Dadabacteria on male *S. thunbergii* and Dependentiae on female *S. thunbergii*. Notably, at low UV-B radiation levels, female *S. thunbergii* presented unique bacterial phyla, while the males did not. Moreover, the number of different genera of bacteria present on the males and females remained unchanged (233 genera of bacteria on the males and 202 genera of bacteria on the females).

#### Effects of increased UV-B radiation on indicator taxa on *S. thunbergii* in different sexes

**Figure 7:**
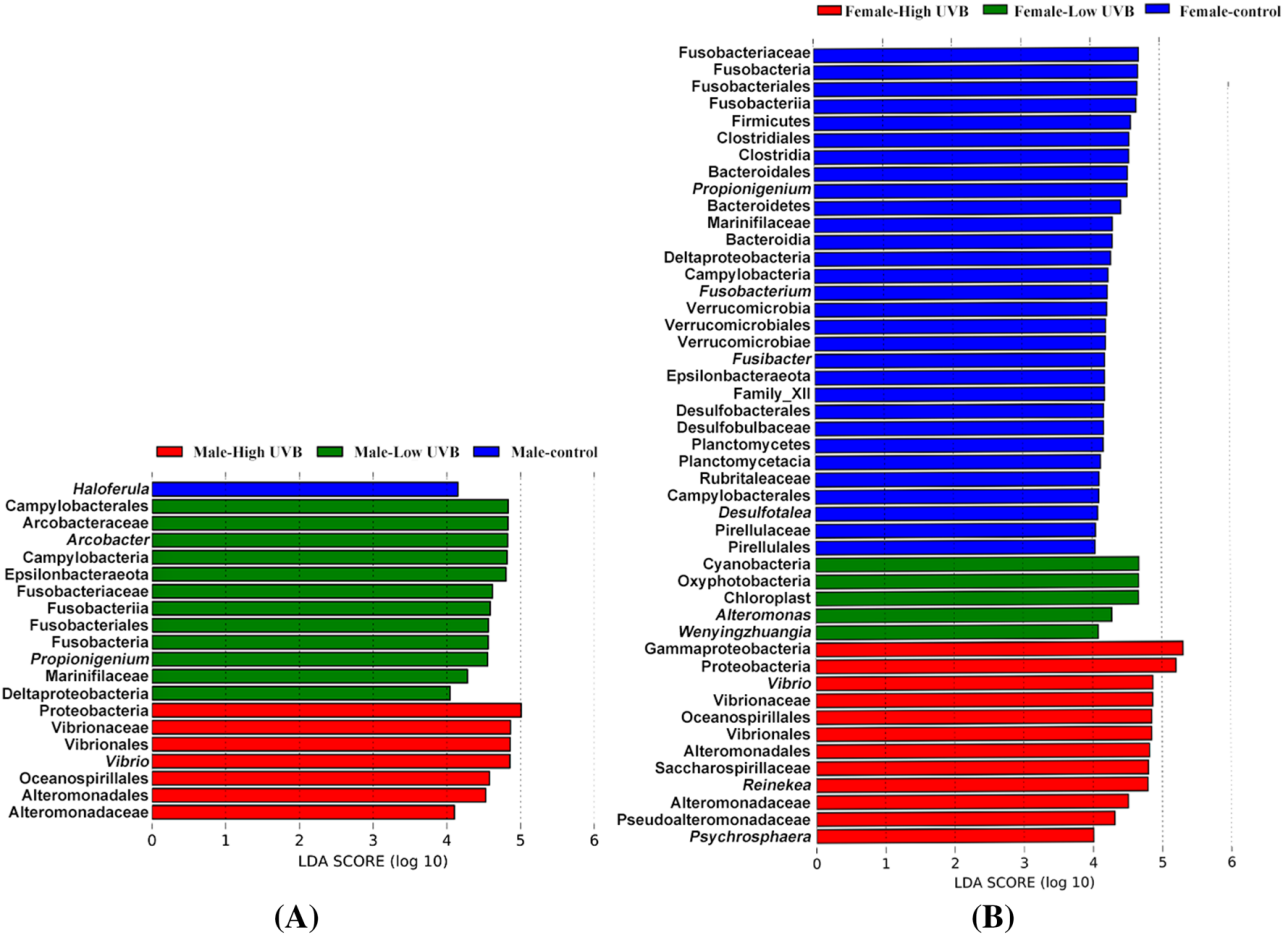
Bacterial species with significant differences in abundance on male (**A**) and female (**B**) *S. thunbergii* under increased UV-B radiation (Forest plot showing taxa whose abundance was significantly different between the control group (blue), low-UV-B radiation group (green) and high UV-B-radiation group (red), as determined using the KW test. LDA score (effect size) indicating significant differences in bacterial taxon abundance (*P* value: Wilcoxon rank-sum test, LDA score > 2.0).

**Figure 8:**
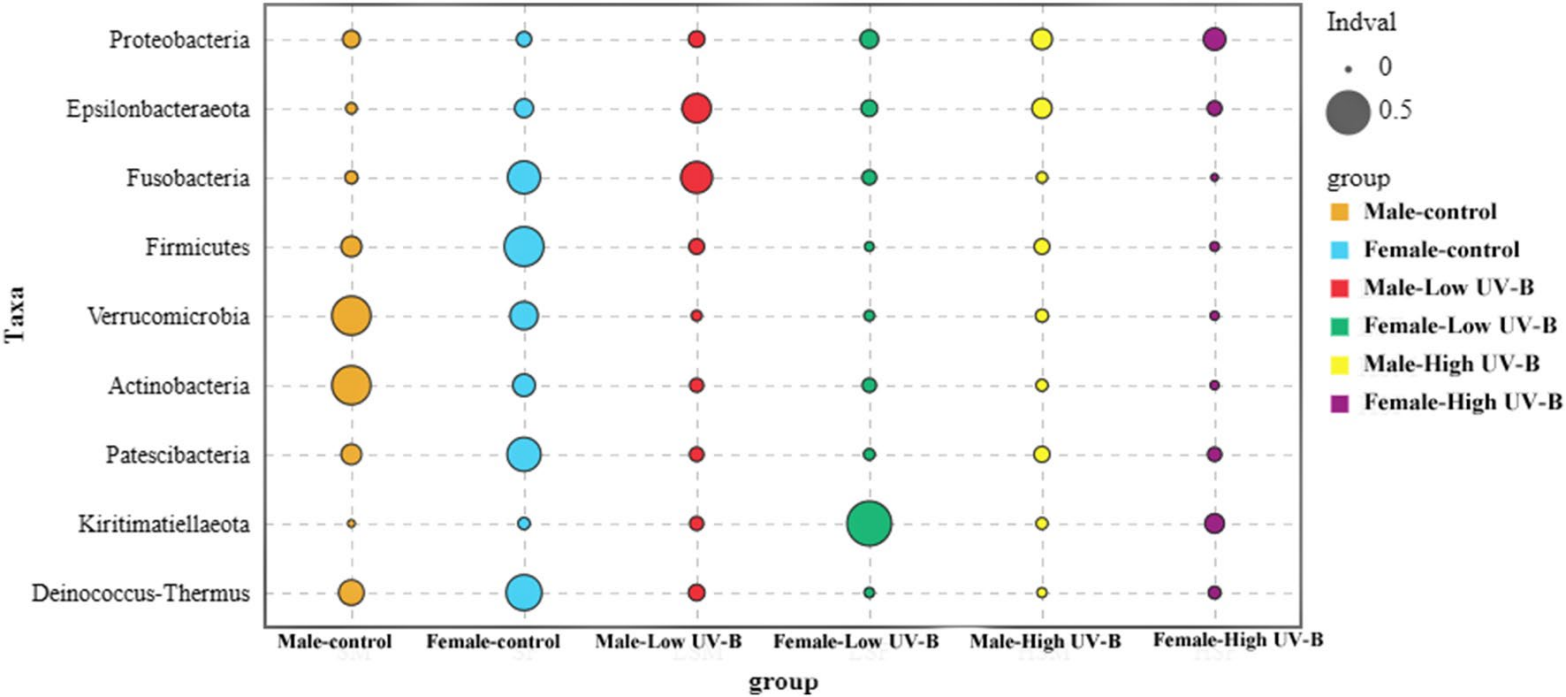
Indicator species (*P* < 0.05) at the phylum level under increased UV-B radiation.

The indicators varied depending on the different sexes of *S. thunbergii* (Fig. 7A, B). At the same time, the indicator values also varied between the males and females (Fig. 8).

There were great differences in indicators, with significant differences occurring between the two sexes. The indicator bacterial groups in each experimental group were different, and the indicator bacteria on female *S. thunbergii* were also significantly more abundant than were those on male *S. thunbergii* (*P* < 0.05) (Fig. 7B).

The indicators with significant differences between male and female *S. thunbergii* were not similar in the control group or in the low-UV-B radiation group, but with the highest UV-B radiation intensity, the change trends were similar (Fig. 8); that is, the indicator values gradually converged and decreased. In the high-UV-B radiation group, the indicator values of epiphytic bacteria on both male and female *S. thunbergii* were similar. Taken together, these results indicated that the differences in abundance between the dominant bacteria decreased and that each indicator bacterium decreased at low abundance.

### Effects of increased UV-B radiation on the predicted functions in *S. thunbergii*

The results of PICRUSt 2 showed that the relative abundance of genes associated with predicted functions in epiphytic bacteria on *S. thunbergii* changed significantly with an increase in UV-B radiation, and this change also differed between samples from male and female *S. thunbergii*.

**Figure 9:**
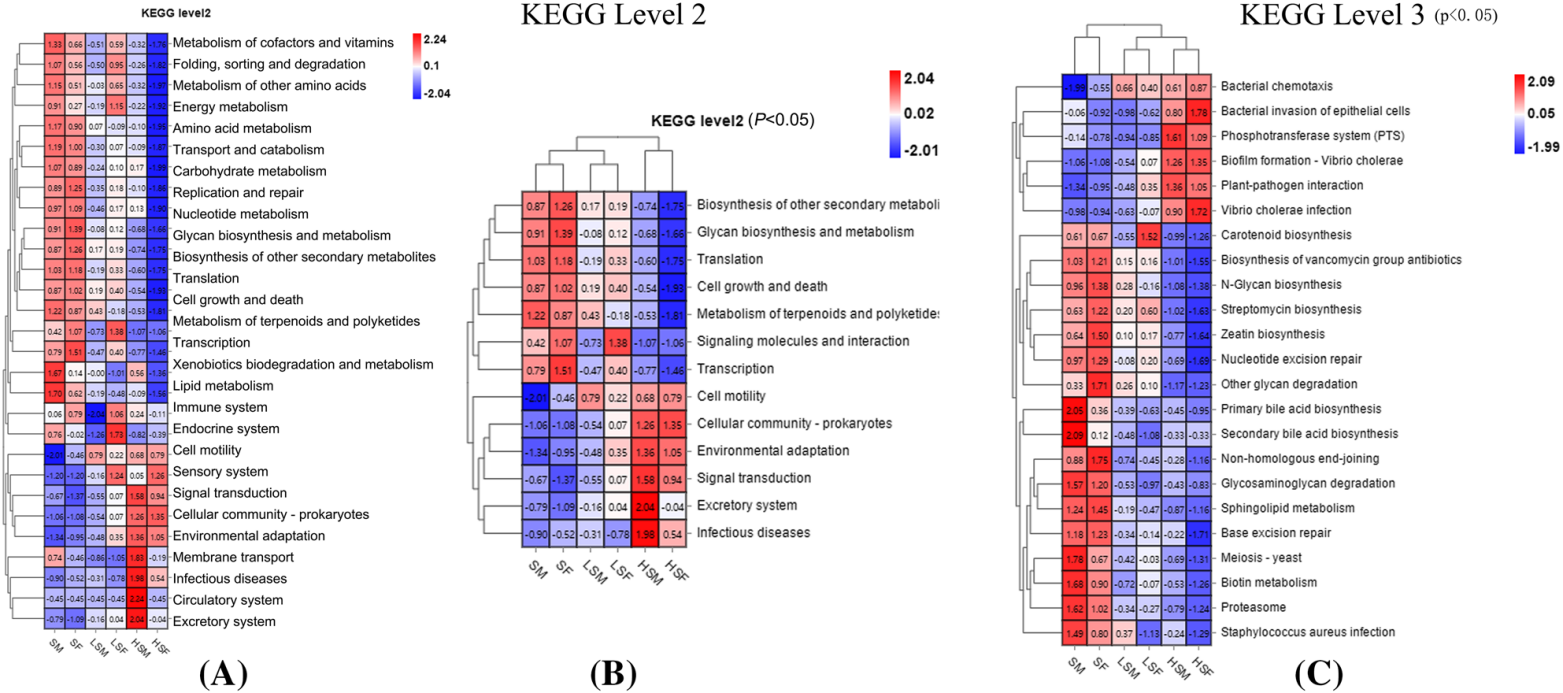
Heatmap of the abundance of genes with functional predictions within epiphytic bacterial communities on female and male *S. thunbergii* under increased UV-B radiation (**A**) Top 34 KEGG level 2 genes with functional predictions, (**B**) genes with significantly different functions (*P* < 0.05) at KEGG level 2, (**C**) genes with extremely significantly different functions (*P* < 0.01) at KEGG level 3, and some genes with significantly different functions (*P* < 0.05), according to the KW rank sum test. For clarity, we use the term “HSM” to refer to “Male-high UV-B”, “LSM” to refer to “Male-low UV-B”, “SM” to refer to “Male-control", “HSF” to refer to "Female-high UV-B”, “LSF” to refer to “Female-low UV-B”, “SF” to refer to "Female-control”.

KEGG level 2 analysis of the predicted genes based on KEGG data indicated that the increased UV-B radiation resulted in obvious changes in the top 29 predicted functions (Fig. 9A), and the greater the dose of UV-B radiation was, the more obvious the change. A heatmap (Fig. 9B) based on the abundance of 13 genes with predicted functions and whose abundance significantly changed (*P* < 0.05) was generated, which showed that the abundance of 7 genes decreased significantly(*P* < 0.05), including genes with functions related to metabolism, biosynthesis, translation, transcription, cell growth and death, signalling molecules and interactions, while the abundance of 6 genes increased, namely, genes with predicted functions in cell motility, environmental adaptation, signal transduction, cellular community-prokaryotes, infectious disease and excretory system. Additionally, 69 of the 179 genes with predicted functions at KEGG level 3 significantly changed (*P* < 0.05) with increasing UV-B radiation. A heatmap (Fig. 9C) based on 23 genes whose abundance significant differed (*P* < 0.01) and some genes with predicted functions and whose abundance significantly differed (*P* < 0.05) was generated, which showed that as the radiation dose increased, the abundance of 11 genes with predicted functions related to metabolism (Streptomycin biosynthesis, N-glycan biosynthesis, glycosaminoglycan degradation, etc.) and 4 genes with predicted functions related to genetic information processing (Proteasome, Non-homologous end-joining, nucleotide excision and base excision repair) decreased significantly(*P* < 0.05). However, the abundance of genes related to phosphotransferase system (PTS) functions related to membrane transport and plant–pathogen interactions related to environmental adaptation increased significantly(*P* < 0.05). In terms of cellular processes, the abundance of genes associated with meiosis in yeast decreased, but that with bacterial chemotaxis and biofilm formation in *Vibrio cholerae* increased(*P* < 0.05). Similarly, regarding the genes with functions associated with *Staphylococcus aureus* infection decreased, the abundance of others (*Vibrio cholerae* infection and bacterial invasion of epithelial cells) increased(*P* < 0.05).

Furthermore, the heatmaps (Fig. 9) showed that the variation in the abundance of genes with predicted functions in response to increasing radiation was not the same in epiphytic bacteria between male and female *S. thunbergii*. When the algae were subjected to low-dose UV-B radiation, genes with predicted functions involving signalling molecules and interactions (KEGG level 2) and genes with predicted functions in carotenoid biosynthesis (KEGG level 3) were less abundant in samples on male *S. thunbergii* than in samples on female *S. thunbergii*. Conversely, the abundance of genes with predicted functions in response to *Staphylococcus aureus* infection from samples on male *S. thunbergii* was higher than that on female *S. thunbergii*. When the algae were subjected to high-dose UV-B radiation, the changes in the abundance of genes with predicted functions in epiphytic bacteria were essentially the same except that the variation in epiphytic bacteria on female *S. thunbergii* was greater than that on male *S. thunbergii*. It is worth noting that under high UV-B radiation, the functions of amino acid metabolism and lipid metabolism of epiphytic bacteria on male *S. thunbergii* are greater than those on female *S. thunbergii*.

## Discussion

In this paper, the 16S rDNA high-throughput sequencing method was used to study the effects of increased UV-B radiation on the epiphytic bacterial communities on male and female *S. thunbergii*. The results showed that under increasing UV-B radiation, the relative abundance of dominant bacteria and indicator bacteria changed significantly, and the changes on the males and females were different.

As a stress factor, UV-B radiation can affect algal epiphytic bacterial communities. Bacteria with strong environmental adaptability became present or increased in abundance. In this study, the abundance of different bacterial groups may have increased or decreased due to differences in sensitivity or resistance to UV-B radiation. For example, among the bacteria with increased abundance in this study, *Alteromonas* bacteria have been reported to show certain resistance to external environmental disturbances^32^. Dobretsov et al.^21^ reported that the relative abundance of *Alteromonas* spp. increased during UV exposure, *Vibrio* bacteria have been reported to grow and reproduce efficiently under environmental stress^33^, and *Reinekea* bacteria have also been reported to be highly adaptable to the environment^34^. In contrast, *Acinetobacter*, whose abundance was reduced, is less adaptable to the environment^35^. Additionally, with the increase in UV-B radiation, unique bacteria became present on *S. thunbergii*. This phenomenon has also been reported in previous studies on bacteria within a maize canopy leaf layer under UV radiation^36^. The presence of unique epiphytic bacteria on *S. thunbergii* under UV-B radiation suggests that sensitive species of epiphytic bacteria die and are replaced by tolerant species or that changes in the algae themselves after exposure to UV radiation support more diverse populations^36^. Based on the abovementioned reasons, UV-B radiation treatment can alter the number of species of epiphytic bacteria on *S. thunbergii.* Notably, the diversity of epiphytic bacteria on *S. thunbergii* decreased under low UV-B radiation but increased under high UV-B radiation. This may be because increased UV-B radiation to some extent alters the microenvironment in which bacteria exist but increases the richness of bacterial communities, which is also consistent with the idea proposed by Lynch and Neufeld^37^ that certain bacteria with a relatively low abundance in a community may help distinguish differences in their environments.

Furthermore, the results showed that after UV-B radiation treatment, the indicator species and indicator values of the epiphytic community of bacteria on *S. thunbergii* changed. There were significant differences in the abundance of some bacteria between the control group and the increased UV-B radiation treatment groups. For instance, *Alteromonas* increased significantly in the treatment group; these bacteria have been reported to have high resistance to UV-B radiation^21^ and have the metabolic ability to degrade a variety of complex polysaccharides and compete for inorganic nutrients^38,39^. *Acinetobacter*, which significantly decreased in the treatment group, has also been associated with the decomposition of cyanobacteria toxins and organic matter^40^. This suggests that UV-B radiation may alter bacterial abundance by affecting the nutrient use ability of epiphytic bacteria on *S. thunbergii*. Moreover, *Vibrio* bacteria, whose abundance significantly increased, have been reported to be pathogens^41^; indeed, many species of *Vibrio* are opportunistic pathogens. This may be due to the reduced health of *S. thunbergii* after UV-B radiation and because the change in physiological state is suitable for the growth of pathogenic bacteria. Notably under high UV-B radiation, the indicator values of bacteria tended to be smaller, the abundance of bacteria also tended to be similar, and the difference decreased. This may be because bacteria have a limited tolerance to stress and because the growth of all bacteria is inhibited under high UV-B radiation. When organisms are under stress, environmental conditions are not amenable to growth. Environmental conditions can lead to stress, which can reduce growth rates, and the same is true of bacteria. For example, Soto et al.^42^ found that the change in adaptability of bacteria under stress is related to the decrease in their growth rate. Besides, Di Cesare et al.^43^ found that, as a method of self-protection, bacteria produce nongrowing cells to improve their adaptability to adverse environments to reduce their sensitivity to harmful environments.

Functional analysis of predicted genes performed by PICRUSt 2 showed that the effects of UV-B radiation on epiphytic bacteria of *S. thunbergii* mainly focused on functions involving metabolism, genetic information processing, infectious diseases and environmental adaptation. The abundance of genes with predicted functions related to metabolism and genetic information of the epiphytic bacteria subjected to UV radiation was significantly lower than that of the control group. These results indicated that increased UV-B radiation had negative effects on the growth and physiology of the epiphytic bacteria. As a mutagen, UV-B radiation mutates DNA redundant, which inevitably therefore gene transcription and protein synthesis and affect various physiological functions of bacteria^44^. In this study, the genes with predicted functions at KEGG level 2 related to genetic information processing, including transcription and translation, as well as the genes with predicted functions at KEGG level 3 involving the proteasome (the proteasome directly affects the recycling of some proteins, including misfolded proteins and many proteins that play an important role in biological activities), nonhomologous end joining (NHEJ), nucleotide excision repair and base excision repair decreased significantly, which indicated that the transcription, translation, and repair of DNA along with protein recycling in epiphytic bacteria were severely damaged by increased UV-B radiation.

At the same time, the genes with predicted functions and that increased in abundance in epiphytic bacteria were mainly related to environmental adaptation. UV-B radiation can result in selective pressure on pathogenic bacteria^45^, causing them to form a protective mechanism to resist UV-B radiation and prevent or reduce the damage to intracellular components^46^. The same is true of the epiphytic bacteria of *S. thunbergii*, which adapted to UV-B radiation stress by increasing the abundance of genes associated with several predicted functions. For example, the abundance of genes with predicted functions in chemotaxis was upregulated, which helped bacteria adapt to the change of chemical substance concentration in the environment^47^. In addition, the genes with predicted function involving the PTS and that increased in abundance can help in the adaptation to the pressure of environmental radiation by controlling the uptake and metabolism of carbohydrates, interfering with the use of nitrogen and phosphorus and affecting the virulence of some pathogens^48^.

The changes in the abundance of genes with predicted functions involving infectious pathogen in response to increased UV-B radiation were interesting. This may be due to the differences in resistance and sensitivity of the different bacteria to radiation and pathogens with high resistance have an abundance of genes with functional predictions. Another reason could be that, UV-B radiation damaged *S. thunbergii* and the ability of *S. thunbergii* to protect itself decreased, which is conducive to bacterial invasion^49^. Huang et al.^46^ found that the expression of pathogenesis-related genes in pathogenic bacteria can significantly decrease during UV-B radiation exposure. In the present study, the abundance of the genes with predicted functions associated with *Staphylococcus aureus* infection also decreased significantly. However, the abundance of genes with other predicted functions associated with *Vibrio cholerae* infection and bacterial invasion of epithelial cells in epiphytic bacteria of *S. thunbergii* increased significantly. The main pathway for pathogens to invade plants is to penetrate the host physiological defensive barrier through invasion and cause host decay via toxins^50^. In this study, the abundance of genes with the predicted function of bacterial invasion of epithelial cells related to bacterial invasion as well as plant–pathogen interactions increased significantly strongly support the above view from the perspective of predicted functions.

On the one hand, after UV-B radiation treatment, the changes in the community of epiphytic bacteria on *S. thunbergii* were due to the direct impact of UV-B radiation on the bacteria themselves. On the other hand, it may be that the response of *S. thunbergii* to UV-B radiation resulted in alterations to the microenvironment on the surface of *S. thunbergii*, which indirectly affected the community of epiphytic bacteria. Due to the difference in the surface microenvironment between male and female algae, the changes in epiphytic bacteria on male and female *S. thunbergii* were different. In this study, at the phylum level, there were only a few dominant bacteria on male and female *S. thunbergii*, such as Bacteroides and Patescibacteria, with the same change trends, and the change trends of more bacteria were different. Most of the bacteria whose abundance significantly changed were related to the growth and development of algae, such as Proteobacteria (at the phylum level), the members of which were related to the growth of algae and account for more than half of the bacteria on both male and female *S. thunbergii*, Epsilonbacteraeota (phylum level), the members of which were related to the synthesis of plant organic matter^51^, Fusobacteria (phylum level), the members of which play important roles in maintaining health^52^, and *Propionigenium* (genus level), the members of which were related to the productivity of algae^53^, and the changes in the abundance of these bacteria between male and female *S. thunbergii* were not consistent. Notably, among the bacteria whose abundance significantly differed, the abundance of Kiritimatiellaeota significantly differed between the males and females. The bacteria of this phylum have been reported to be related to the degradation of polysaccharides, Sun found soluble sugars served as the regulated metabolites in male *S. thunbergii* responding to UV-B radiation and Lu et al.^12^ also found that the content of soluble sugars significantly increased in male *S. thunbergii* under UV-B radiation. It suggests that the physiological and biochemical properties of *S. thunbergii* of the different sexes may influence the composition of bacteria to some extent. Therefore, we hypothesized that the different responses of epiphytic bacteria placed on male and female *S. thunbergii* to UV-B radiation were mainly caused by the metabolic difference between the sexes of host algae. The changes in the metabolism of male and female *S. thunbergii* could lead to the changes in the metabolism of algal epiphytic bacteria, resulting in the difference in the response of epiphytic bacteria communities on different sexes of the host algae to UV-B radiation. Further studies need to be performed to verify this hypothesis using the high-throughput sequencing combined with metabolome analysis.

Besides, the changes in the abundance of genes with predicted functions of epiphytic bacteria on male and female *S. thunbergi*i were different. For example, at low doses of radiation, the genes with predicted functions involving *Staphylococcus aureus* infection were enriched in epiphytic bacteria on male *S. thunbergii*, while the genes with predicted functions involving carotenoid biosynthesis were enriched in epiphytic bacteria on female *S. thunbergii.* At high UV-B radiation doses, the types of and changes in the genes of predicted functions and that increased in abundance were essentially no different in epiphytic bacteria between male and female *S. thunbergii*. However, the genes with predicted function and that decreased in abundance in epiphytic bacteria on female *S. thunbergii* decreased more than those did on male *S. thunbergii,* indicating that high-dose UV-B radiation does more harm to females than to males. Previous studies have shown that under exposure to UV-B radiation, the amino acid and energy metabolism^12–14^ of male *S. thunbergii* were enhanced compared with female *S. thunbergii*, which might help the males behaved better ability to resist and defend the UV-B stresses. Chen et al.^25^ reported that the chlorophyll content and osmotic regulatory ability of male mulberry seedlings were higher than those of female mulberry seedlings, and the antioxidant enzymes of male mulberry seedlings were more responsive to UV-B radiation. However, more experiments are needed to verify whether the epiphytic bacteria on male *S. thunbergii* were more resistant to UV-B radiation than were those on female *S. thunbergii*.

Knowledge concerning the effects of epiphytic bacteria on macroalgae in response to environmental stress is still limited due to the limitations of complex mechanisms and research methods^23^. The method used to study epiphytic bacteria on macroalgae is an important factor in identifying and understanding the diversity and function of bacteria on the surface of macroalgae^54^. Our results indicated that it was feasible to use a high-throughput method to study the response of epiphytic bacterial communities to environmental changes and provide new idea for experimental methods of macroalga–bacterium interactions.

## Conclusion

16S rDNA high-throughput sequencing was used to analyse the effects of increased UV-B radiation on communities of epiphytic bacteria on male and female *S. thunbergii*. The results indicated that the increase in UV-B radiation had a direct impact on epiphytic bacteria on *S. thunbergii* and that the relative abundance of dominant bacteria, indicator species and indicator values changed significantly. Moreover, the changes in epiphytic bacteria on male and female *S. thunbergii* were different, which shows that changes in the microenvironment of the host *S. thunbergii* after UV-B radiation indirectly affects the epiphytic bacterial community. The predicted functional results indicated that with an increase in UV-B radiation, the abundance of genes whose functions are related to metabolism and genetic information processing decreased, and the abundance of genes whose functions are related to the environmental adaptation of bacteria increased significantly; however, the abundance of some genes with functions involving infectious pathogen increased, and that of some decreased. Taken together, our results revealed that 16S rDNA high-throughput sequencing is a feasible way to directly investigate the effects of increased UV-B radiation on epiphytic bacteria of *S. thunbergii* and can reveal the changes in bacterial communities after UV-B radiation. This study may provide a reference for clarifying the response of epiphytic bacteria on macroalga to enhanced UV-B radiation and reveale that the thinning of the global ozone layer will affect the community structure and function of the epiphytic bacteria of marine macroalgae, which will also lead to the destruction of their symbiotic relationship with the algae host, and may affect marine biogeochemical cycle.

## Methods

### Sampling site and sample collection

*S. thunbergii* samples were collected from the rocky intertidal zone (36°N, 120°E) in Qingdao (Shandong, China) in July, 2020. Samples of *S. thunbergii* of both sexes (with a similar habitat, that were growing well, approximately 10 cm in height and 25 g in weight, no spots caused by disease or insect pests) were selected and placed in sterilized sealed bags. There are significant differences in morphology and internal structure between male and female *S. thunbergii* during the reproductive period^55,56^. Male receptacles were slender and smooth, while the females were short and coarse. The sex of *S. thunbergii* were identified according to the morphological features of the receptacles and then samples of similar numbers of males and females were collected and was taken back to the laboratory for further examination of the internal structure under a microscope (Nikon H600L, Tokyo, Japan). Female receptacles have many oval egg cells; while males do not, which could allow us to further determine the *S. thunbergii* sex.

### UV-B radiation treatment

After additional sand grains, sediment, small herbivores and epiphytic miscellaneous algae on the surface of the algal bodies were removed, *S. thunbergii* was cultivated and allowed to acclimated after repeated washes with sterilized natural seawater. The acclimation period was carried out in a glass incubator (120 cm × 70 cm × 50 cm) with 15 ± 0.5 °C at the photosynthetically active radiation (PAR) intensity of 150 μmol·m^−2^·s^−1^, 12 L/12 D (light: dark cycle). *S. thunbergii* of similar size, of similar colour and displaying good growth were randomly chosen and subjected with UV-B radiation.

In the experiment, an irradiation system simulating increased UV-B radiation on macroalgae in the intertidal zone^57^ was used for artificially imposing stress treatments under laboratory conditions. The sterilized natural seawater was used and replaced once every day. The seawater for experiment with the salinity of 31 ± 1 and pH of 8.0 ± 0.2 was taken from macroalgal grown places and stored temporarily in the laboratory for a period of time after collection. The light was provided by fluorescent tubes (Philips TL-D, 36 W) and UV-B broadband lamps (Philips TL 40 W/12 RS) wrapped with cellulose acetate film. The radiation system needed to be continuously run for 3 days before the formal experiment to enhance the stability of membrane filtration. The light intensity between each radiation treatment group was adjusted by changing the number of light tubes and the distance between the tubes and *S. thunbergii*. The UV-B radiation intensity was measured with a UV spectroradiometer (Beijing Normal University, Beijing, China). According to the average intensity of UV-B radiation at sampling sites (2.29 W·m^−2^ at noon on sunny days in the summer in coastal areas of Shinan district, Qingdao, Shandong, China) and the preliminary results, one control group (UV-B radiation intensity of 0, normal fluorescent tube irradiation) and two radiation treatment groups (low UV-B radiation intensity (1.5 W·m^−2^) and high UV-B radiation intensity (2.5 W·m^−2^) were included, and each group had 8 replicates (4 males/4 females). The experiment lasted for 5 days, with UV-B radiation applied for 8 h·day^−1^ ^58^. Since the experimental algae began to wither and die on the 4th day (When the color of *S. thunbergii* become darker, the leaves fall off, the algae become soft and rotten, and the seawater in the incubator is turbid, and it can be considered that the algae is dead), the epiphytic bacteria of all groups were isolated and measured on the 3rd day (Table 1).

**Table 1:** Experimental groups of the effects of increased UV-B radiation on epiphytic bacteria of male and female *S. thunbergii.*

Experimental groups	PAR intensity (μmol·m^−2^·s^−1^)	UV-B intensity (W·m^−2^)	Samples	Group name
Control group	150	0	8 (4 Male/4 Female)	Male-control/Female -control
Low UV-B intensity treatment	150	1	8 (4 Male/4 Female)	Male- Low UV-B/Female -Low UV-B
High UV-B intensity treatment	150	2.5	8 (4 Male/4 Female)	Male-High UV-B/Female-High UV-B

Only 22 samples were actually analyzed, because DNA was unable to be extracted from 2 samples from *S. thunbergii* (1 female and 1 male) in the control group.

Henceforth, "high", "low" and " control " were used to distinguish the different UV-B radiation intensities, and "Male-high UV-B”, “Male-low UV-B”, and “Male-control" as well as "Female-high UV-B”, “Female-low UV-B”, and “Female-control" were used to distinguish the male and female experimental groups, respectively.

### Acquisition of bacteria from the surface of *S. thunbergii*

Bacteria on the surface of *S. thunbergii* were obtained as previously described^59,60^, with slight modifications. A 25 g sample of *S. thunbergii* at 3 days after treatment was weighed and placed in a sterile triangular flask, to which 70 mL of sterile phosphate-buffered saline (PBS) buffer solution (1 mmol·L^−1^) was added; afterward, the flask was sealed with sterilized film. The flask was subsequently shaken (200 r·min^−1^) for 30 min at room temperature to obtain a suspension of epiphytic bacteria isolated from the surface of *S. thunbergii*.

### High-throughput sequencing of the 16S rDNA

In the past, studies on the effects of different stresses on algal epiphytic bacteria were mostly conducted in aseptic systems and isolation cultures and tried to identify epiphytic bacteria related to macroalgae. However, because sterile experimental systems are difficult to obtain and culturable bacteria constitute less than 1% of the bacteria present in nature, in actual algal environments, bacteria do not exist in isolation^61^; thus, these methods are cumbersome, and the information obtained is not accurate or comprehensive. In this paper, high-throughput sequencing, which is relatively fast and has a relatively low cost and workload^23^, was used to extract DNA samples directly from algae.

On the ultra-clean platform, the bacterial suspension obtained in was filtered through sterile gauze to remove any impurities, and then the bacteria were filtered and collected on a 0.22 µm filter membrane using a vacuum filtration device. DNA was extracted from these membranes using an E.Z.N.A. Stool DNA Kit (Omega Bio-tek, USA) following the manufacturer’s instructions. At the same time, a negative control was used to determine the contamination from the DNA Kit. The 16S rDNA V3-V4 region was amplified via PCR using 341F (5′-CCTACGGGNGGCWGCAG-3′) and 806R (5′-GGACTACHVGGGTATCTAAT-3′) primers. The purified amplicons were subsequently sequenced (PE250) on the Illumina Hiseq 2500 platform according to standard protocols by Guangzhou Genedenovo Biotechnology Co., Ltd.

### Data analysis

Data analysis was conducted according to the method described by Mathai^60^. Paired-end 16S sequences were assembled using FLASH (v.1.2.7). According to fqtrim (V 0.94), clean tags were acquired by quality filtering of the raw reads, after which chimeric sequences were filtered using Vsearch software (v2.3.4). DADA2 was further used to produce amplicon sequence variant (ASV) tables from the filtered data. QIIME 2 software^62^ was used to analyse the α diversity and β diversity, and R software was used to plot the results graphically. Welsh’s test and the Kruskal–Wallis (KW) test were used to evaluate the significance of the α diversity indices, *a*nd the difference in the β diversity was tested using the analysis of similarities (ANOSIM) test*.* BLAST *w*as used for sequence alignment, and each representative sequence was annotated via the SILVA (Release 132) and Greengenes databases (v 13.5), which were used as reference taxonomic databases^63^. Taxonomic summaries were performed by calculating the relative abundance across samples and normalizing to 100%. Rarefaction curves were plotted for each sample based on the ASV table to compare epiphytic bacterial species abundance among *S. thunbergii* samples^64^. Linear discriminant analysis effect size (LefSe) analysis was subsequently performed using LefSe software^65^ in conjunction with the KW rank sum test and the Wilcoxon rank sum test (linear discriminant analysis (LDA) score > 2). Indicator analysis was used to calculate the indicator value of each species in each group based on the abundance and frequency of occurrence of species in the sample. The R labdsv package was used to calculate the indicator value of species with an abundance value > 0 and a total proportion > 0.1% in each group within a comparison group. Cross-validation was then used for statistical testing to obtain p values. The results are displayed in a bubble chart. PICRUSt 2 software^66^ and the Kyoto Encyclopedia of Genes and Genomes (KEGG) pathway database^67–69^ were used for functional gene annotation and analysis^70^. After functional predictions of each group, cluster analysis was carried out using a heatmap. The nonparametric factorial KW rank sum test was used to determine the taxa whose abundance significant differed.

## Supplementary Information

Supplementary Information 1.

## Data Availability

The datasets generated and analysed during the current study are available in the NCBI Sequence Read Archive (SRA) repository under BioProject ID PRJNA759186 (Biosample accession number SAMN21157271: Male-high UV-B: SRR17154927, SRR17154928, SRR17154947, and SRR17154948; Male-low UV-B: SRR17154929-SRR17154932; Male-control: SRR17154933, SRR17154938, SRR17154949, and SRR17154950; Female-high UV-B: SRR17154934-SRR17154937; Female-low UV-B: SRR17154939-SRR17154942; Female-control: SRR17154943-SRR17154946).
