# Supplementary material for: Effects of UV-B radiation on epiphytic bacterial communities on male and female Sargassum thunbergii
Source: Sci Rep. 2023 Mar 9;13:3985. doi: 10.1038/s41598-022-26494-3 (PMC9998616; doi:10.1038/s41598-022-26494-3)
Supplement: Supplementary file 1 — Supplementary Information 1. [file 41598_2022_26494_MOESM1_ESM.docx]

**Effects of** **UV-B radiation on** **epiphytic bacterial communities on male and female** ***Sargassum thunbergii***

**Jing Wang^1^, Zhibo Yang^1^, Peiyao Lu^1^, Yan Sun^1^, Song Xue^1^,** **Xuexi Tang^1,2*^, Hui Xiao^1,2*^**

^1^ College of Marine Life Sciences, Ocean university of China, 266003 Qingdao, China

^2^ Laboratory for Marine Ecology and Environmental Science, Qingdao National Laboratory for Marine Science and Technology, 266000 Qingdao, China

*** Correspondence:**Xuexi Tang
tangxx@ouc.edu.cn

Hui Xiao
xiaohui@ouc.edu.cn

# Supplementary Figures


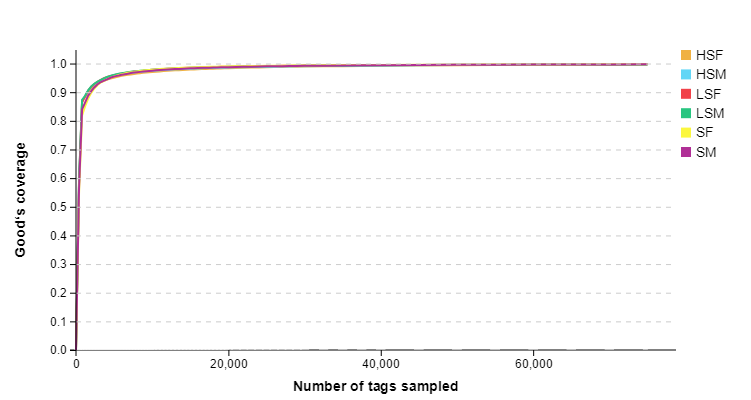


**Supplementary Fig S1** Sequencing depth of the bacterial community on *S. thunbergii* under increased UV-B radiation.


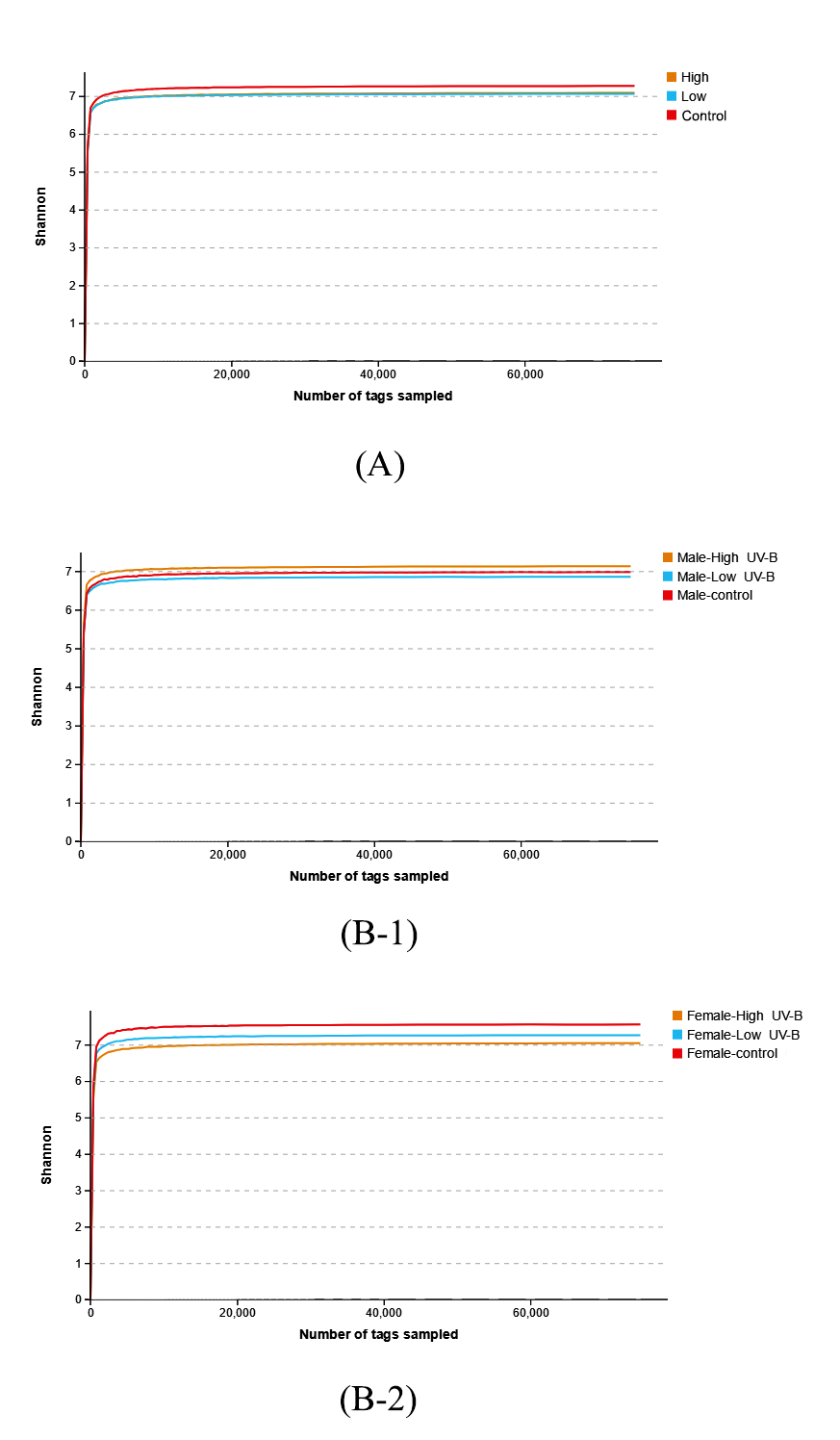


**Supplementary Fig S2** α diversity of epiphytic bacterial communities on *S. thunbergii* under increased UV-B radiation of (A) different intensities and (B) for different sexes: (B-1) male *S. thunbergii* (B-2) female *S. thunbergii*

# Supplementary Tables

**Supplementary Table S1.** α diversity p value of epiphytic bacterial communities on male and female *S. thunbergii* (according to Welsh’s test and the KW test)

| **Diffs** | ***P*-value** | **test** |
| --- | --- | --- |
| Male-High UV-B VS Male-Low UV-B | 0.529746 | Welsh’s test |
| Male-High UV-B VS Male-control | 0.99249 | Welsh’s test |
| Male-Low UV-B VS Male-control | 0.529035 | Welsh’s test |
| Male-High UV-B, Male-Low UV-B and Male-control | 0.579238 | Kru-Wall test |
| Female-High UV-B and Female Low UV-B | 0.01164* | Welsh’s test |
| Female-High UV-B and Female-control | 0.180285 | Welsh’s test |
| Female-Low UV-B and Female-control | 0.358707 | Welsh’s test |
| Female-High UV-B, Female-Low UV-B and Female-control | 0.034872* | Kru-Wall test |
| Male VS Female | 0.688523 | Welsh’s test |
| High, Low and Control | 0.124078 | Kru-Wall test |

Note: Asterisks (*) indicates significant difference (*P* < 0.05)

**Supplementary Table S2.** p value of the β diversity of epiphytic bacteria on male and female *S. thunbergii* (according to ANOSIM tests)

| **Diffs** | **R-value** | ***P*-value** | **significant** |
| --- | --- | --- | --- |
| High vs Low vs Control | 0.5687 | 0.001 | ** |
| Female-High UV-B vs Female-Low UV-B vs Female-control | 0.94 | 0.001 | ** |
| Male-High UV-B vs Male-Low UV-B vs Male-control | 0.27 | 0.075 |  |
| Female-High UV-B vs Male-High UV-B vs Female-Low UV-B vs Male- Low UV-B vs Female-control vs Male-control | 0.5055 | 0.001 | ** |

Note: Asterisks (**) indicate extremely significant (*P* < 0.01)

**Supplementary Table S3.** Changes in the relative abundance of the top 12 bacteria at the phylum level on male and female *S. thunbergii* under increased UV-B radiation

| **Phylum** | **Control (%)** | **Low (%)** | **High (%)** | **Male-control (%)** | **Male-Low UV-B (%)** | **Male-High UV-B (%)** | **Female-control (%)** | **Female-Low UV-B (%)** | **Female-High UV-B (%)** |
| --- | --- | --- | --- | --- | --- | --- | --- | --- | --- |
| **Proteobacteria** | 47.5980^b^  ±1.85 | 52.7107^b^  ±2.75 | 71.308^a^  ±1.55 | 52.8512^b^  ±2.50 | 48.1186^b^  ±4.20 | 67.625^a^  ±2.60 | 42.3448^b^  ±1.20 | 57.3028^b^  ±1.30 | 74.9918^a^  ±0.50 |
| **Bacteroidetes** | 15.9405^a^  ±1.55 | 15.7254^a^  ±1.50 | 12.3458^b^  ±0.57 | 14.6551^a^  ±1.85 | 16.1610^a^  ±1.55 | 12.3788^a^  ±0.89 | 17.2259^a^  ±1.25 | 15.2898^a^  ±1.45 | 12.3128^b^  ±0.25 |
| **Epsilonbacteraeota** | 7.138^a^  ±1.30 | 13.0047^a^  ±1.15 | 8.9401^a^  ±0.27 | 4.2736^a^  ±1.55 | 17.7112^a^  ±1.85 | 10.9173^b^  ±0.44 | 10.0025^a^  ±1.05 | 8.2982^a^  ±0.44 | 6.9628^b^  ±0.10 |
| **Fusobacteria** | 6.4970^a^  ±1.60 | 6.6192^a^  ±1.02 | 1.3925^b^  ±0.7 | 2.7719^a^  ±2.35 | 9.7998^a^  ±1.75 | 2.0728^b^  ±0.45 | 10.2221^a^  ±0.85 | 3.4386^a^  ±0.29 | 0.7122^b^  ±0.95 |
| **Cyanobacteria** | 3.314^a^  ±1.05 | 5.6330^a^  ±1.675 | 1.5463^a^  ±0.93 | 5.8152^a^  ±1.85 | 0.9888^a^  ±1.85 | 1.2677^a^  ±1.85 | 0.8129^a^  ±0.25 | 10.2773^a^  ±1.50 | 1.8249^b^  ±0.01 |
| **Firmicutes** | 6.1042^a^  ±0.90 | 1.7521^b^  ±0.3 | 1.7476^b^  ±0.33 | 3.7690^a^  ±1.05 | 2.5009^a^  ±0.25 | 2.4936^a^  ±0.56 | 8.4393^a^  ±0.74 | 1.0034^b^  ±0.35 | 1.0017^b^  ±0.10 |
| **Verrucomicrobia** | 4.6449^a^  ±0.15 | 0.8619^b^  ±0.25 | 0.9139^b^  ±0.15 | 5.5482^a^  ±0.25 | 0.8665^a^  ±0.44 | 1.2385^a^  ±0.25 | 3.7416^a^  ±0.04 | 0.8573^b^  ±0.05 | 0.5893^b^  ±0.05 |
| **Planctomycetes** | 4.2037^a^  ±0.57 | 1.3236^b^  ±0.03 | 0.3865^c^  ±0.05 | 5.1349^a^  ±0.85 | 1.5913^a^  ±0.20 | 0.4519^a^  ±0.05 | 3.2725^a^  ±0.28 | 1.0560^a^  ±0.04 | 0.3210^b^  ±0.05 |
| **Actinobacteria** | 1.4268^a^  ±1.18 | 0.4871^b^  ±0.21 | 0.2825^b^  ±0.04 | 1.8925^a^  ±0.25 | 0.4824^a^  ±0.12 | 0.3665^a^  ±0.05 | 0.9610^a^  ±1.85 | 0.4918^a^  ±0.03 | 0.1986^a^  ±0.02 |
| **Patescibacteria** | 0.8995^a^  ±0.02 | 0.3090^b^  ±0.02 | 0.3984^b^  ±0.02 | 0.6225^a^  ±0.01 | 0.3693^a^  ±0.02 | 0.4327^a^  ±0.03 | 1.1766^a^  ±0.20 | 0.2487^b^  ±0.02 | 0.3640^b^  ±0.01 |
| **Spirochaetes** | 0.1999^b^  ±0.02 | 0.8497^a^  ±0.02 | 0.1236^b^  ±0.01 | 0.0472^a^  ±0.01 | 0.8425^a^  ±0.01 | 0.1264^a^  ±0.01 | 0.3525^a^  ±0.02 | 0.8569^a^  ±0.02 | 0.1208^b^  ±0.01 |
| **Tenericutes** | 0.7617^a^  ±0.01 | 0.2045^a^  ±0.01 | 0.2316^a^  ±0.01 | 1.3920^a^  ±0.01 | 0.1633^a^  ±0.01 | 0.3299^b^  ±0.01 | 0.1314^a^  ±0.01 | 0.2457^a^  ±0.01 | 0.1332^b^  ±0.01 |
| **bSum** | 98.7281 | 99.4808 | 99.6173 | 98.7732 | 99.5953 | 99.7017 | 98.6831 | 99.3663 | 99.5330 |

Note: Different letters (a, b, c) denote significant differences (Wilcoxon rank-sum test, P < 0.05) between groups: The same symbol indicated that there were no statistically significant differences between groups; the different symbols indicated that the differences were statistically significant between groups.

**Supplementary Table S4.** Changes in the relative abundance of the top 12 bacteria at the genus level on male and female *S. thunbergii* under increased UV-B radiation

| **Genus** | **Control (%)** | **Low (%)** | **High (%)** | **Male-control (%)** | **Male-Low UV-B (%)** | **Male-High UV-B (%)** | **Female-control (%)** | **Female-Low UV-B (%)** | **Female-High UV-B (%)** |
| --- | --- | --- | --- | --- | --- | --- | --- | --- | --- |
| ***Arcobacter*** | 6.7053^b^  ±0.60 | 12.5202^a^  ±1.66 | 8.6128^b^  ±0.25 | 3.8787^c^  ±0.70 | 17.331^a^  ±2.30 | 10.410^b^  ±0.35 | 9.5318^a^  ±0.50 | 7.7087^a^  ±1.01 | 6.8153^a^  ±0.15 |
| ***Vibrio*** | 0.9452^c^  ±0.25 | 3.7990^b^  ±0.96 | 16.0049^a^  ±2.85 | 0.5864^c^  ±0.33 | 3.1132^b^  ±1.57 | 16.0205^a^  ±2.85 | 1.3040^b^  ±0.16 | 4.4849^b^  ±0.34 | 15.989^a^  ±0.44 |
| ***Reinekea*** | 0.3066^c^  ±0.12 | 1.6219^b^  ±1.08 | 10.7365^a^  ±0.88 | 0.3222^b^  ±0.11 | 0.8444^b^  ±0.72 | 8.3413^a^  ±1.32 | 0.2911^b^  ±0.12 | 2.3994^b^  ±1.44 | 13.131^a^  ±0.43 |
| ***Propionigenium*** | 3.5922^a^  ±0.31 | 5.0201^a^  ±0.38 | 0.4963^b^  ±0.15 | 0.6265^b^  ±0.26 | 8.1291^a^  ±0.44 | 0.7697^b^  ±0.26 | 6.5579^a^  ±0.35 | 1.9110^a^  ±0.31 | 0.2229^b^  ±0.03 |
| ***Alteromonas*** | 0.0551^b^  ±0.01 | 2.6335^a^  ±0.62 | 2.6065^a^  ±0.28 | 0.0605^a^  ±0.01 | 1.3966^a^  ±0.63 | 1.4630^a^  ±0.42 | 0.0497^b^  ±0.01 | 3.8703^a^  ±0.06 | 3.7500^a^  ±0.14 |
| ***Wenyingzhuangia*** | 1.1644^b^  ±0.13 | 2.8965a  ±0.52 | 0.9941^b^  ±0.04 | 0.9286^b^  ±0.20 | 2.2626^a^  ±0.53 | 0.7953^b^  ±0.05 | 1.4002^b^  ±0.06 | 3.5304^a^  ±0.51 | 1.1929^b^  ±0.03 |
| ***Acinetobacter*** | 3.3772^a^  ±0.45 | 0.9240^b^  ±0.28 | 0.7477^b^  ±0.13 | 5.0215^a^  ±0.67 | 1.6557^b^  ±0.55 | 1.2525^b^  ±0.15 | 1.7328^a^  ±0.23 | 0.1922^b^  ±0.01 | 0.2429^b^  ±0.10 |
| ***Fusibacter*** | 2.0359^a^  ±0.12 | 0.5445^b^  ±0.14 | 0.8625^b^  ±0.06 | 0.7079^b^  ±0.22 | 0.8673^b^  ±0.26 | 1.2957^a^  ±0.02 | 3.3639^a^  ±0.02 | 0.2216^b^  ±0.02 | 0.4294^b^  ±0.10 |
| ***Polaribacter_4*** | 0.6162^b^  ±0.01 | 1.1740^a^  ±0.01 | 1.0893^a^  ±0.01 | 0.7620^a^  ±0.01 | 1.3854^a^  ±0.01 | 1.1964^a^  ±0.01 | 0.4703^a^  ±0.01 | 0.9627^a^  ±0.01 | 0.9823^a^  ±0.01 |
| ***Psychromonas*** | 0.1578^b^  ±0.01 | 1.4574^a^  ±0.01 | 1.0875^a^  ±0.01 | 0.0847^a^  ±0.01 | 1.0268^a^  ±0.01 | 0.9672^a^  ±0.01 | 0.2310^b^  ±0.01 | 1.8880^a^  ±0.01 | 1.2077^b^  ±0.01 |
| ***Shewanella*** | 0.1431^c^  ±0.01 | 0.6588^b^  ±0.01 | 1.5528^a^  ±0.01 | 0.0389^b^  ±0.01 | 0.4598^b^  ±0.01 | 2.0456^a^  ±0.01 | 0.2472^a^  ±0.01 | 0.8578^a^  ±0.01 | 1.0601^a^  ±0.01 |
| ***Neptunomonas*** | 0.3102^b^  ±0.01 | 1.7973^a^  ±0.01 | 0.1675^c^  ±0.01 | 0.3937^b^  ±0.01 | 2.0442^a^  ±0.01 | 0.2479^b^  ±0.00 | 0.2267^b^  ±0.00 | 1.5504^a^  ±0.01 | 0.0872^b^  ±0.01 |
| **Sum** | 19.4091 | 35.0471 | 44.9584 | 13.4115 | 40.5168 | 44.8052 | 25.4067 | 29.5774 | 45.1116 |

Note: Different letters (a, b, c) denote significant differences (Tukey HSD, P < 0.05) between groups: The same symbol indicated that there were no statistically significant differences between groups; the different symbols indicated that the differences were statistically significant between groups.

| Classification | UV-B radiation | Unique taxa on Male *S. thunbergii* | Unique taxa on Female *S. thunbergii* | Common taxa to both *S. thunbergii* sex |
| --- | --- | --- | --- | --- |
| Phythum | Control | 2 | 4 | 22 |
|  | Low UV-B | 3 | 3 | 19 |
|  | High UV-B | 1 | 2 | 23 |
|  | Sum | 0 | 5 | 24 |
| Class | Control | 4 | 8 | 41 |
|  | Low UV-B | 7 | 7 | 36 |
|  | High UV-B | 4 | 7 | 41 |
|  | Sum | 3 | 11 | 45 |
| Genus | Control | 66 | 45 | 255 |
|  | Low UV-B | 45 | 34 | 260 |
|  | High UV-B | 53 | 36 | 252 |
|  | Sum | 75 | 47 | 292 |

**Supplementary Table S5.** Changes in the number of phyla, classes, and genera common to both *S. thunbergii* sex and sex-unique bacteria under increased UV-B radiation
